# Supplementary material for: Social networks predict the life and death of honey bees
Source: Nat Commun. 2021 Feb 17;12:1110. doi: 10.1038/s41467-021-21212-5 (PMC7889932; doi:10.1038/s41467-021-21212-5)
Supplement: Supplementary file 1 — Supplementary Information [file 41467_2021_21212_MOESM1_ESM.pdf]

Supplementary Information for 'Social networks predict the life and death of honey bees'

Benjamin Wild[1*]†, David M Dormagen[1]†, Adrian Zachariae[2]†,
Michael L Smith[3,4,5], Kirsten S Traynor[1,6], Dirk Brockmann[2,7],
Iain D Couzin[3,4,5], Tim Landgraf[1*]

[1] Department of Mathematics and Computer Science, Freie Universität Berlin, Berlin, Germany.
[2] Robert Koch Institute, Berlin, Germany.
[3] Department of Collective Behaviour, Max Planck Institute of Animal Behavior, Konstanz, Germany.
[4] Centre for the Advanced Study of Collective Behaviour, University of Konstanz, Konstanz, Germany.
[5] Department of Biology, University of Konstanz, Konstanz, Germany.
[6] Global Biosocial Complexity Initiative, Arizona State University, Tempe, USA.
[7] Institute for Theoretical Biology, Humboldt University Berlin, Berlin, Germany.

[*] Corresponding authors; b.w@fu-berlin.de, tim.landgraf@fu-berlin.de
† Equal contribution

Supplementary Note 1: What is network age?

Supplementary Note 1.1: Contributions of the interaction types on network age

To understand the relative contributions of the different interaction types on network age, we can visualize the coefficients of the linear mapping from the spectral factors learned by the CCA (or PCA, for the network age PCA variant). This also allows us to compare the three dimensions of network age 3D against each other and the PCA variant.

There is a strong collinearity between the spectral decomposition factors derived from the different interaction matrices (because the interaction themselves are strongly correlated, e.g. the number of proximity interactions is correlated with the number of trophallaxis interactions). Therefore, directly interpreting the CCA weights would yield inaccurate results and we first have to decorrelate the spectral embeddings. We apply a PCA to the spectral embeddings, similar to the network age PCA variant, but retain all principal components to which we then apply the CCA. We then have two linear mappings, one from the CCA and one from the PCA. Sequential linear mappings can be combined into one, allowing us to attribute the coefficients from the CCA to the spectral factors, i.e. to measure to what extent each factor contributes to network age:

For spectral factors $F \in \mathbb{R}^{N \times S}$ and the linear mappings $M^{PCA} \in \mathbb{R}^{S \times S}$ and $M^{CCA} \in \mathbb{R}^{S \times 3}$, we calculate the contributions $C \in \mathbb{R}^{S \times 3}$: $C = S^{-1}|M^{PCA}M^{CCA}|$. S is the total number of spectral factors (see Methods: Network age - CCA). N is the number of bee days, i.e. we have one set of spectral factors for each individual per day. We use the absolute value of the contributions because the sign of the spectral factors is not informative.

We see that all interaction types contribute to network age: The first dimension of network age is mainly based on the first spectral factor of each interaction type, with the proximity interactions being slightly more

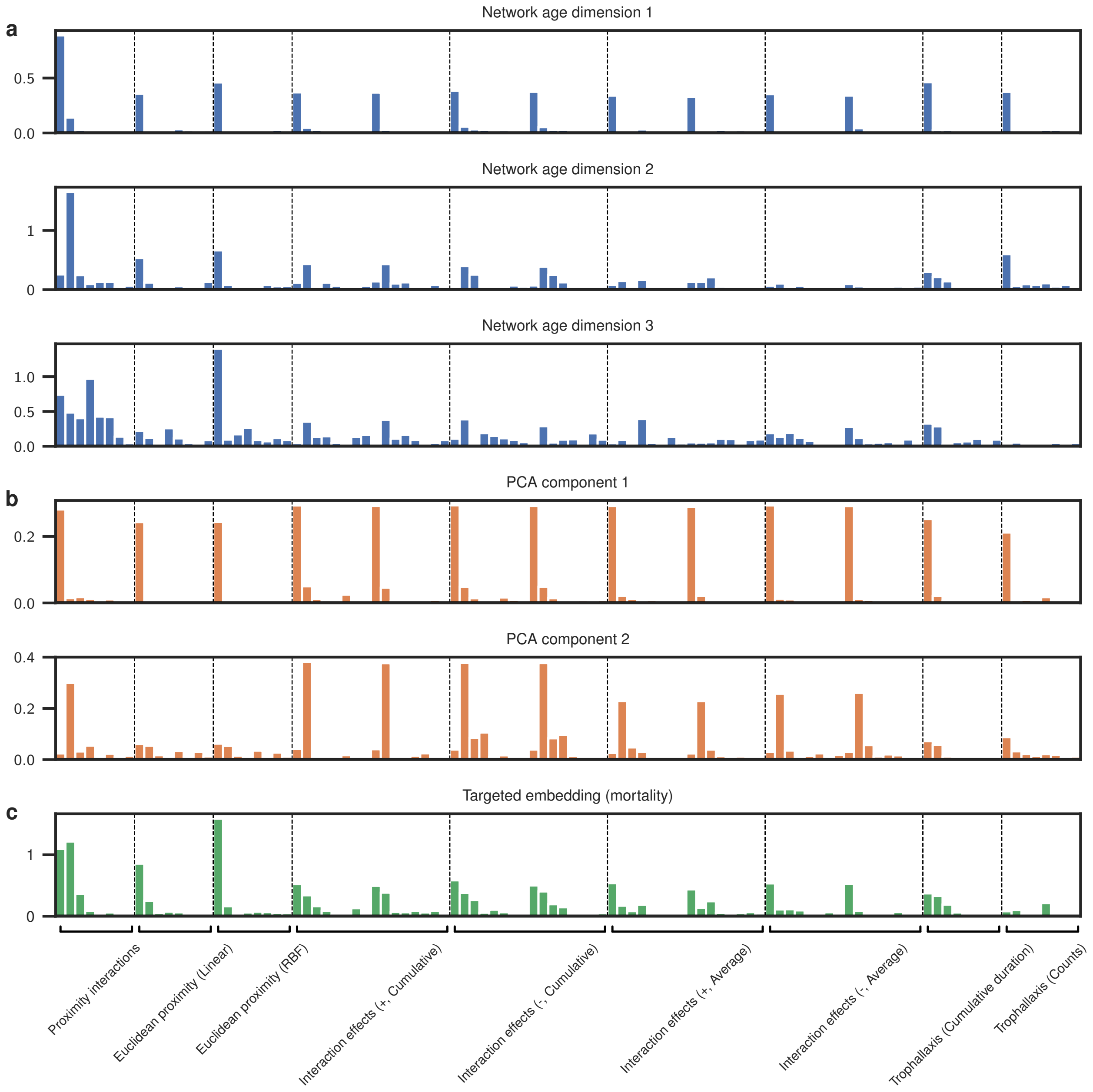

Supplementary Figure 1: **Relative contributions of spectral factors to network age.** For each spectral factor derived from the interaction matrices for the different interaction types, we calculate the relative contributions for the multidimensional variant of network age (**a**, blue), network age PCA (**b**, orange), and the targeted embedding for mortality (**c**, green). A high value signifies that the corresponding factor has a large impact on network age.

important. Therefore, the first factor of the spectral decomposition alone is already a good representation of task allocation. We see a similar pattern in the first principal component of the PCA, which also explains the similarly high predictiveness of the PCA variant regarding task allocation. In contrast, the second dimension of network age is very different from the PCA's second dimension. This shows that the CCA can select the relevant information from the spectral embeddings.

For the targeted embedding of a bee's mortality, we find that the CCA selects a more diverse set of factors than the first dimension of network age and the first component of the PCA. This shows that different aspects

of the social network are relevant for different research questions and explains why the PCA version of network age is much worse when predicting mortality (see Supplementary Table 1).

Supplementary Note 1.2: Location heatmaps

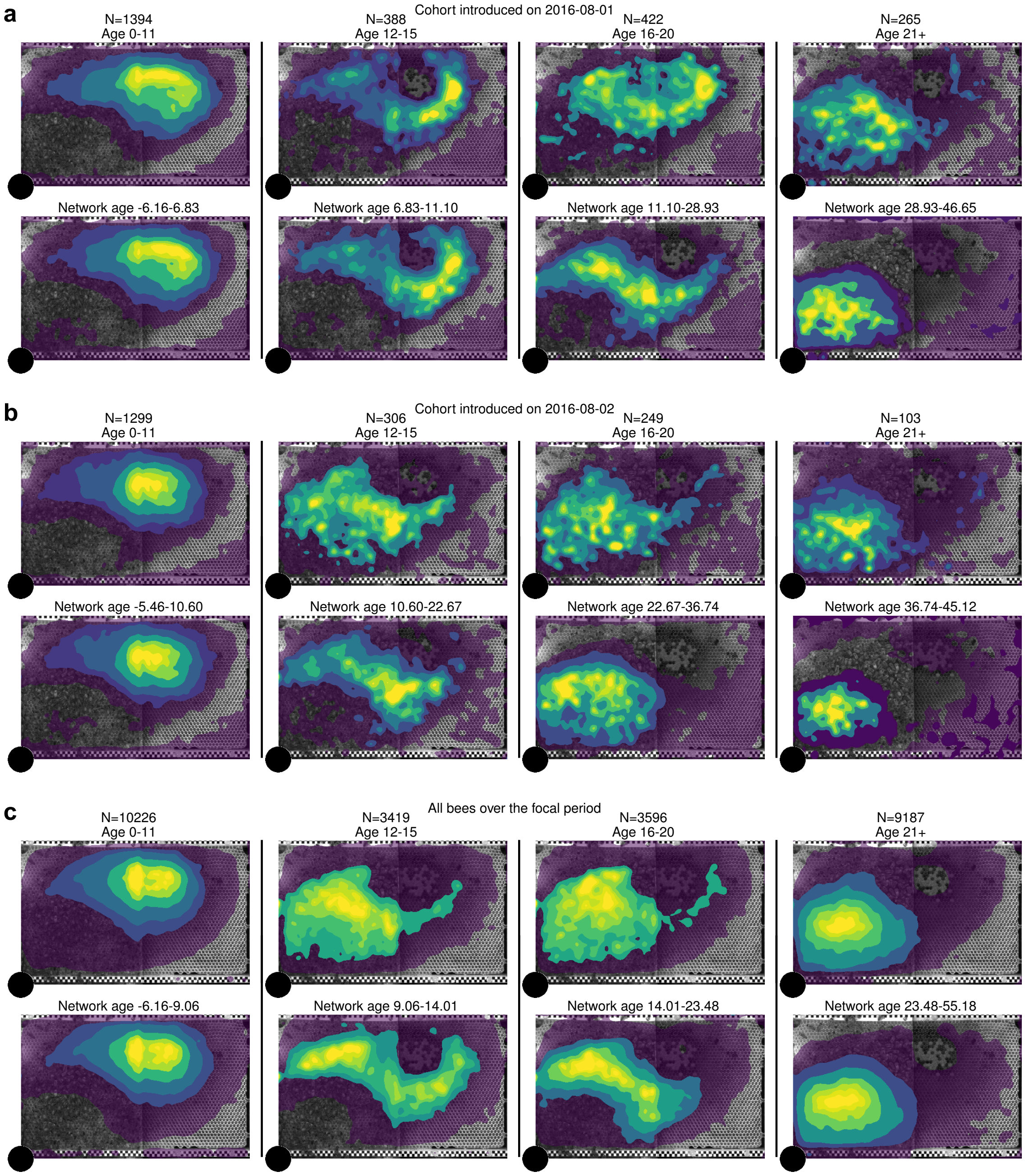

Supplementary Figure 2: **Spatial heatmaps of bees grouped by biological age and network age. a** Cohort of 2016-08-01 (N=123 bees). **b** Cohort from 2016-08-02 (N=119 bees). **c** All bees over the focal period (N=1912 bees). The shaded areas depict density percentiles (brightest to darkest: 99%, 97.5%, 95%, 80%, 70%, 20%).

To visually assess how distinctive biological age and network age are with respect to the spatial distribution of individuals, we computed location heatmaps for single-age cohorts tracked over the experimental window (25 days). For each individual within the cohort, we collected their daily biological age, network age, and

their positions on the comb (as in Methods: Nest area mapping and task descriptor). Positional data for each day (a 'bee day') was then assigned to a heatmap as follows: For the age heatmaps, we manually defined age intervals based on the age-thresholds given by Seeley[1] , with an additional split for middle-aged bees for which we observed a high variance. We then assigned bee days according to these thresholds based on the bees' ages on the different days. The network age thresholds for the plots have been set in a way to keep the number of bee days the same for the two plots in each column and can, therefore, differ for different cohorts. For example, the cohort introduced on 2016-08-01 consisted of 123 bees. In the heatmap for age 0-11, there would be N=1394 bee days over the focal period in which the bees were below 12 days old (some bees having died). Then, we calculated the first network age thresholds in such a way, that they would also include 1394 bee days.

The shaded areas depict density percentiles (brightest to darkest: 99%, 97.5%, 95%, 80%, 70%, 20%).

See Supplementary Figures 2a and 2b for heatmaps showing the distributions of different example cohorts that we introduced at the beginning of the focal period and Supplementary Figure 2c for all bees.

Supplementary Note 1.3: Network age distributions over time

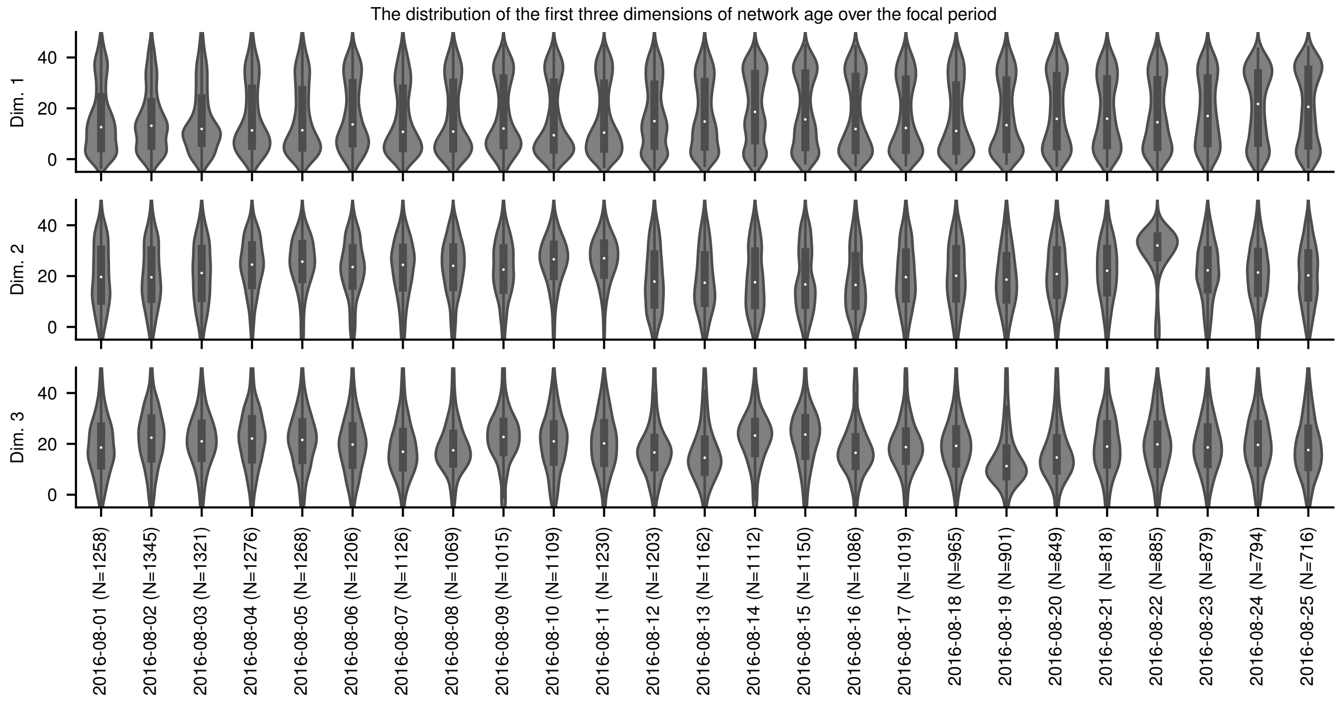

Supplementary Figure 3: **Violin plots of network age distributions over the focal period.** The first three dimensions of network age are depicted. Each violin comprises one dimension of the network age values of all individuals on a given day (the number of individuals on each day is given in the x-axis labels; boxes: center dot, median; box limits, upper and lower quartiles; whiskers, 1.5x interquartile range).

While the network age distribution stays within similar bounds over time, we find that the shape of the distribution (e.g. the proportion of individuals in the lower vs higher percentiles) changes significantly over the experimental window, likely reflecting changes in task allocation (e.g. more individual engaged in brood care initially and more foragers after most brood cells are capped). See Supplementary Figure 3 for distributions over time.

We find no strong evidence of correlations of these changes in distribution with external factors such as the weather, but this may be due to the limited size of the recording window and is an interesting direction for potential future research.

Supplementary Note 2: Network age correctly identifies task allocation

Supplementary Note 2.1: Spatial separation of bees with similar network age

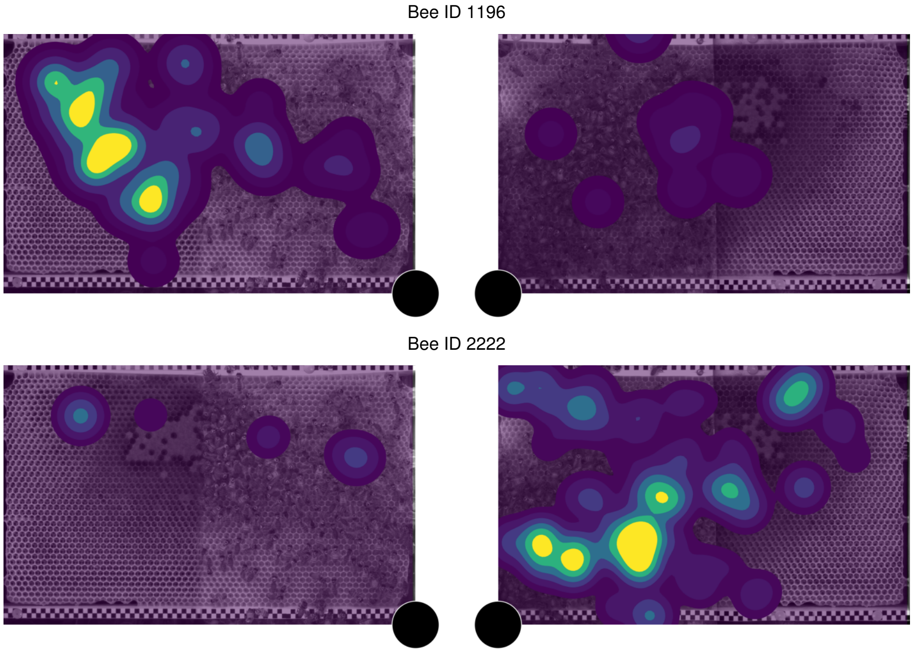

Supplementary Figure 4: **Two bees on 2016-08-05 with similar network ages despite occupying different sides of the comb.** The bee with the ID 1196 is 22 days old and has a network age of 15.09. Her heatmap consists of 1189 samples, each corresponding to one minute on the focal day. The bee with the ID 2222 is 8 days old and has a network age of 14.34. Her heatmap consists of 1284 samples, each corresponding to one minute on the focal day. The black circle marks the location of the entrance. The shaded areas depict density percentiles (brightest to darkest: 99%, 97.5%, 95%, 80%, 70%, 20%).

While the network age of bees is correlated with their locations, we do find examples of bees that have approximately the same network age but occupy different locations on the comb (see Supplementary Figure 4 for an example). We also see that, while two bees having a similar network age is correlated with an increase in proximity interactions, there are many bees with close network ages that do not encounter each other inside the hive (see Supplementary Figure 5).

Supplementary Note 2.2: Effect of subsampling bees, the dimensionality of spectral embeddings, and only using proximity information on task prediction accuracy

Network age can be computed with a much smaller proportion of marked bees, hence allowing one to study much larger colonies, or reducing the effort for marking individuals. To quantify the effect of sparse sampling, we perform the following analysis. We subsample the individuals entering the analysis and compute interaction networks only for those selected individuals, i.e. only interactions between individuals

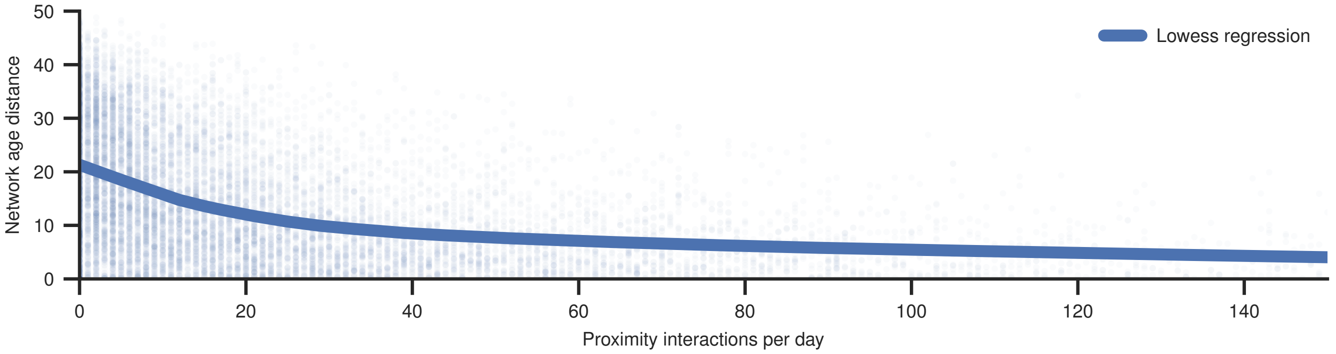

Supplementary Figure 5: **The relationship between distance in network ages and number of proximity interactions for pairs of bees.** Close network ages generally mean more interactions. However, there are many individuals with a close network age and very few interactions. Each point indicates one pair of individuals on one day. The line is given by a lowess regression.

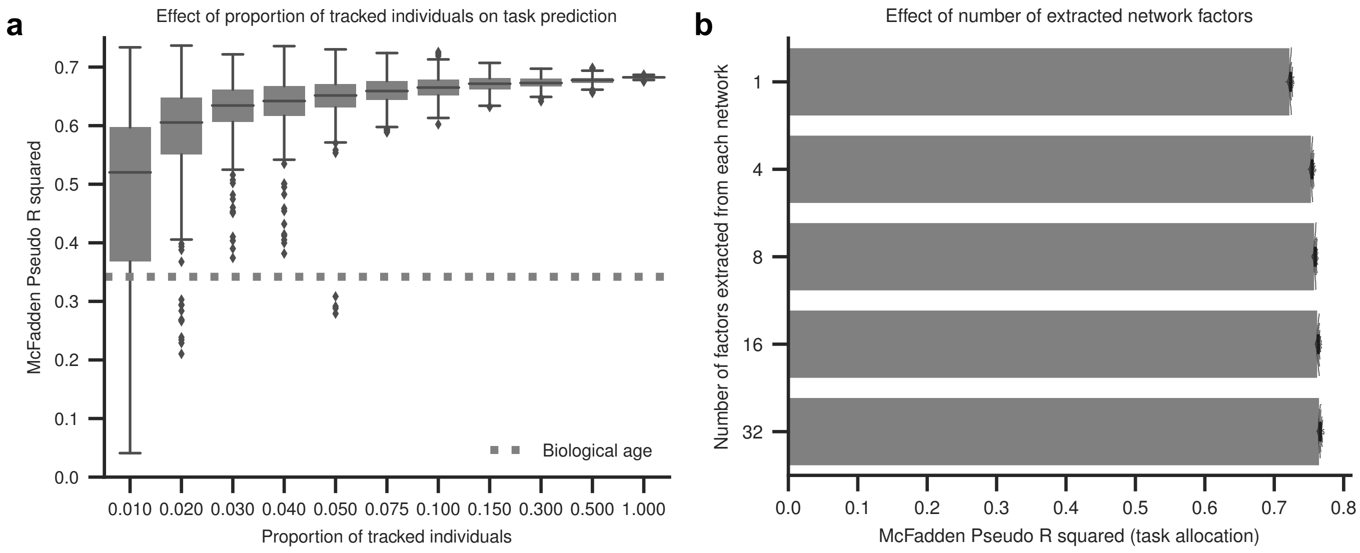

Supplementary Figure 6: **Effects of subsampling the individuals and the number of spectral factors from each interaction network on the task allocation prediction. a** McFadden's R^2 for different fractions of subsampled individuals. While having all bees individually marked and tracked yields the highest median score, tracking only 5% of the individuals yields comparable results when drawing a representative subset. Each box comprises N=128 bootstrap runs (center line, median; box limits, upper and lower quartiles; whiskers, 1.5x interquartile range; points, outliers). **b** The influence of the number of dimensions per interaction network extracted using Spectral embedding before applying the CCA on the task allocation prediction scores (using network age 3D). While more dimensions enable the CCA to extract more relevant information, the overall improvement is small. The bars show the mean of N=128 bootstrap samples per dimensionality and the error bars a bootstrap sampled 95% CI of the mean.

of this subset are recorded. We then compute spectral factors and network age as described in Methods: Network age - CCA. We find that network age is remarkably robust in this setting. With 5% of the bees tagged, the proportion in variance explained using network age is comparable to the case of using all individuals. Even with only 1% of the individuals used in the analysis, network age still is a better predictor of task allocation than biological age. This suggests that a much larger colony could be analyzed with this

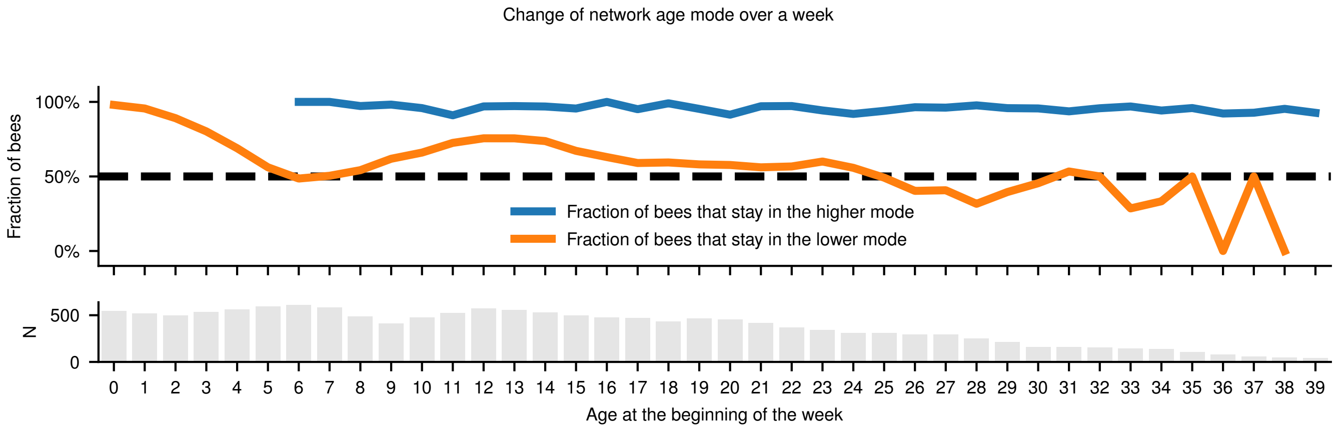

Supplementary Figure 7: **The consistency of network age cluster over the course of a week.** (Top) We considered every individual and day (first day) for which we had data one week later (seventh day). We sorted them by their biological age on the first day (x-axis). Bees that were assigned to the higher network age cluster on the first day make up the blue line, bees from the lower cluster are depicted by the orange line. The y-axis value of each line is the fraction of bees that are still assigned to the same cluster seven days later. A high proportion of bees that are assigned to the higher cluster stay there over the course of one week (blue line). There are more bees that transition from the lower cluster to the higher one, regardless of age (orange line). (Bottom) The number of data points (orange and blue combined) for each age.

method without additional effort, as long as a representative subset of the bees is tracked. See Supplementary Figure 6a for the influence of different subsampling fractions on the results.

Likewise, we also find that the number of spectral factors per interaction mode matrix does not have a strong effect on the proportion of variance explained by the network age extracted from those factors. Using more factors is computationally more expensive and produces statistically better embeddings. However, network age computed using 32 factors explains task allocation only marginally better compared to network age computed using 4 factors (see Supplementary Figure 6b).

We also evaluate variants of network age that can be derived using only the spatial information of detected individuals. To that end, we exactly follow the procedure outlined in Methods: Network age - CCA, but discard all interaction types except for either spatial proximities ('Euclidean proximity' variant, see 'Euclidean proximity networks' in Methods: Social networks) or interaction events derived from spatial proximity ('Proximity events' variant, see 'Proximity interaction network' in Methods: Social networks). We find that network age derived from all interaction types outperforms these proximity based variants in terms of task allocation, but the variant based on proximity events is only marginally worse (see Methods: Task prediction models and bootstrapping) and could be used in future studies if only proximity data is available.

Supplementary Note 2.3: Change of network age mode over a week

We analyze how many bees that are assigned to either of the network age modes on day X are assigned to the same cluster on day X+7 (clusters calculated as in Methods: Quantifying when bees first split into distinct network age modes). Most of the bees in the upper cluster are also in the upper cluster one week later (mean=93% 95% CI [77%, 100%], N=3698 bee days).

Bees in the lower cluster tend to stay there less (mean=56% 95% CI [0%, 96%], N=10 927 bee days) depending on their age (see Supplementary Figure 7).

Supplementary Note 2.4: Variability of task prediction accuracy over time

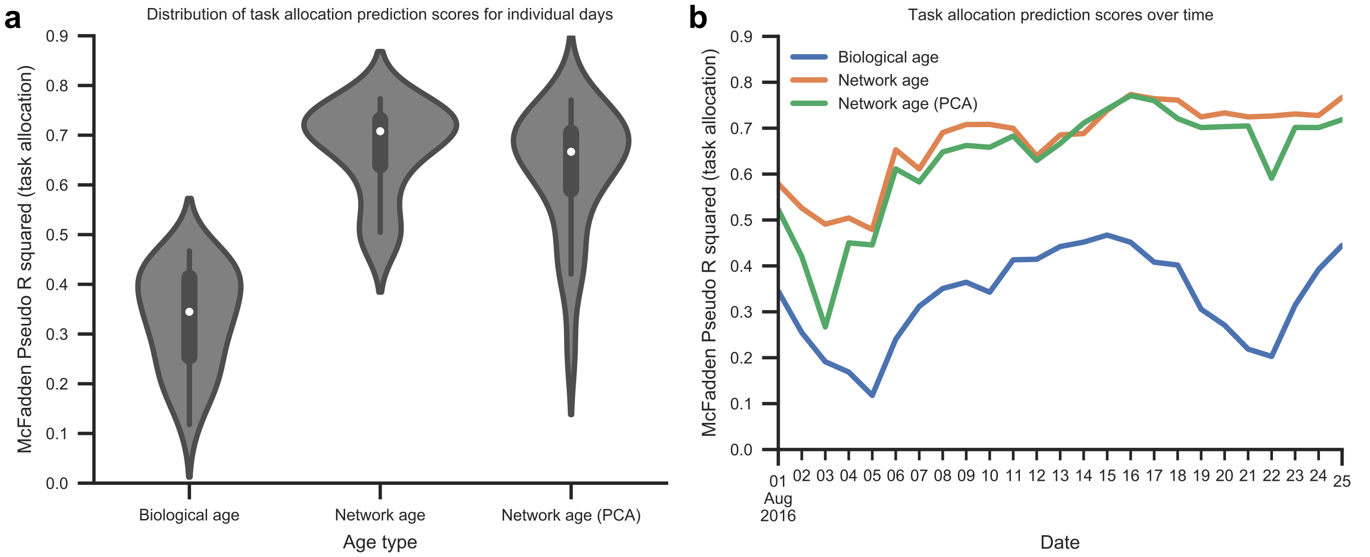

Supplementary Figure 8: **Stability of task allocation prediction over time. a** The distribution of McFadden's R^2 for task allocation prediction for the different days using either network age, the unsupervised network age (PCA), or biological age as predictors (N=25 individual days; boxes: center dot, median; box limits, upper and lower quartiles; whiskers, 1.5x interquartile range). **b** The same data points plotted over time.

The task allocation prediction scores are not constant over time for biological age, network age, and the unsupervised (PCA) variant of network age. Here, we show McFadden's Pseudo R^2 scores for individual days. While there is variance in the predictability using network age, we note that on all dates, the models based on network age yield a higher score than the models using biological age. On all days, the unsupervised PCA variant is more predictive than biological age with comparably small differences to the CCA variant. See Supplementary Figure 8 for the distribution and timeline.

Supplementary Note 3: Network age predicts an individual's behavior and future role in the colony

Supplementary Note 3.1: Probability of dying over the course of a week

We have shown that network age is more predictive of the remaining days to live than biological age (see Methods: Prediction of other behavior-related measures). Here we directly compare biologically young but functionally old bees with biologically old but functionally young bees. To define biologically young and old, we orientate ourselves at the caste thresholds proposed by Seeley[1]. To define functionally young and old we use the same thresholds for network age as shown in the heatmaps of Fig. 3a, because they occupy distinct locations on the comb and therefore hold different functional roles within the colony. However, these exact thresholds are not important as both the effect strength and the significance are high.

We sort the individuals into the two groups based on their network age and biological age. To not count an individual twice, we only consider the last date that a bee was seen in each group. We calculate the probability of dying P as the fraction of bees of each group we do not consider alive after one week (see Methods: Bayesian lifetime model for more information about the death date of a bee). We calculate a confidence interval by drawing 1000 bootstrap samples for each group.

Biologically young but functionally old bees (biological age < 11 days, network age > 30) have a probability of dying over the course of a week of P=80.6% (bootstrap 95% CI=[74.1%, 87.1%], N=139). Biologically old but functionally young bees (biological age > 20, network age < 15) have a significantly lower probability of dying of P=42.1% (bootstrap 95% CI=[37.4%, 47.2%], N=390; χ^2 test of independence $p \ll 0.001$ N=529).

Supplementary Note 3.2: Time spent in task-associated locations as a predictor

To evaluate how well the time spent in the measured task-associated locations itself is able to predict an individual's behavior and future role in the colony, we derive two representations of this spatial information and use them as predictors in the same way as network age (as described in Methods: Prediction of other behavior-related measures, Methods: Future predictability) for predicting mortality and movement patterns.

We use the task descriptor described in Methods: Nest area mapping and task descriptor either directly as the independent variables in the regression models ('Location (4D)' in Supplementary Table 1a) or a 1D representation of this descriptor derived using PCA ('Location (1D)' in Supplementary Table 1a). The 4D descriptor is not directly comparable to network age because of the higher dimensionality, but serves as an upper bound on the information contained in the task descriptor. In terms of dimensionality, the 1D representation can directly be compared to network age.

We find that this spatial information is a better descriptor of an individual's behavior than age, but significantly less informative when compared to network age (Supplementary Table 1d). See Supplementary Table 1 for an overview of the scores and effect sizes.

Supplementary Figures and Tables

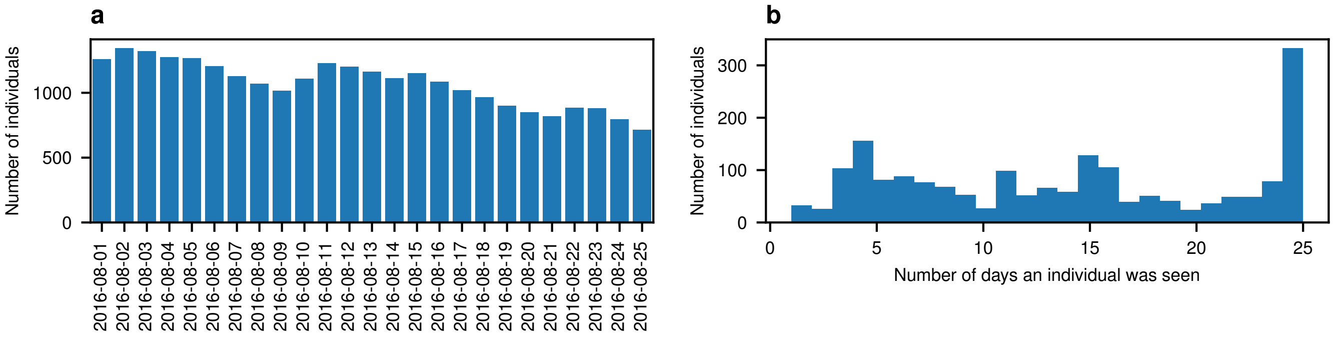

Supplementary Figure 9: **Number of bees observed during our focal period.** **a** The number of bees per day. Days on which we introduced new bees are indicated by a rise in the population. **b** The number of days any single bee was observed during our focal period. The peak at 25 indicates bees that lived throughout the whole period.

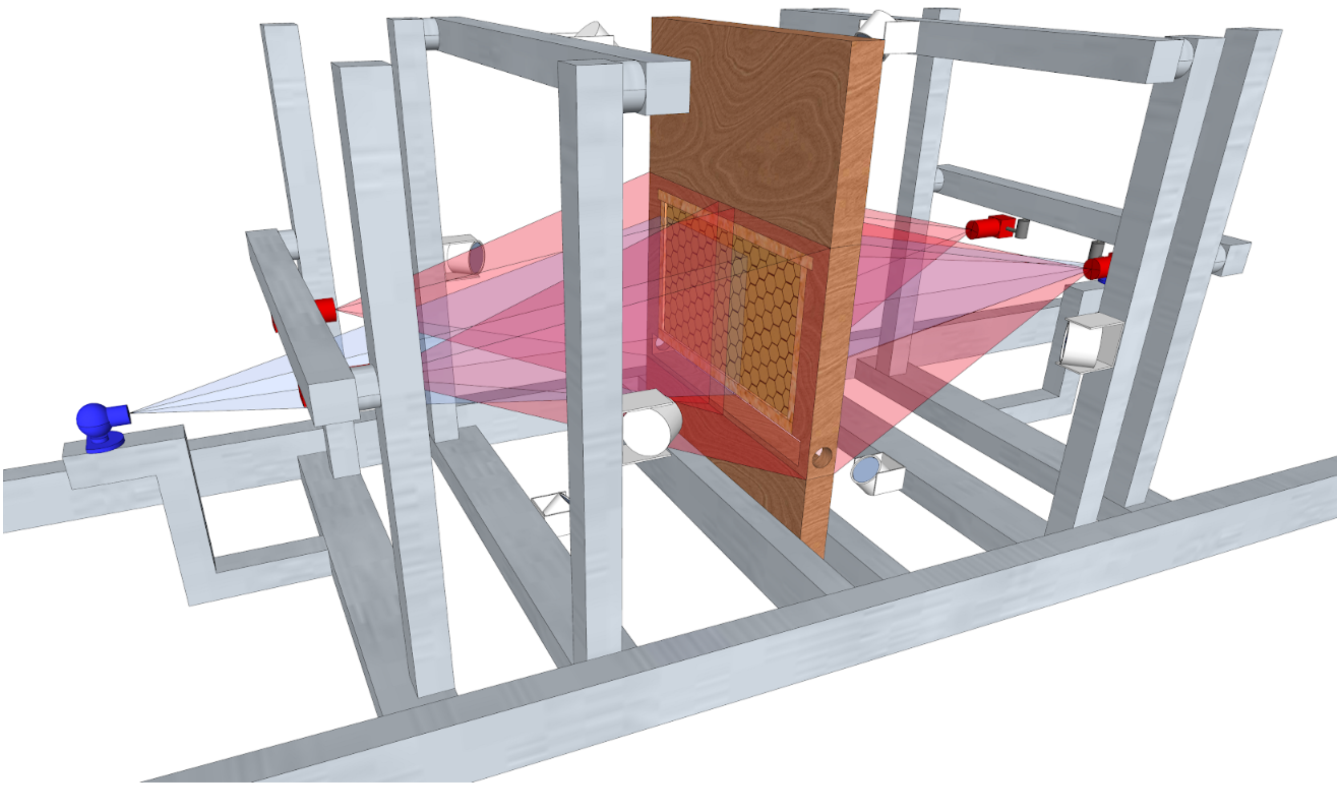

Supplementary Figure 10: **Recording setup** A one-frame observation hive stands in the center of the rig, two high-res cameras (red), and one low-res camera (blue) observe each side of the hive. The low-res cameras only observe the dance floor. Four infrared flashlights were used per side (synchronized to the high-res cameras), constant red lighting illuminated the dance floor. Adapted with permission from[2].

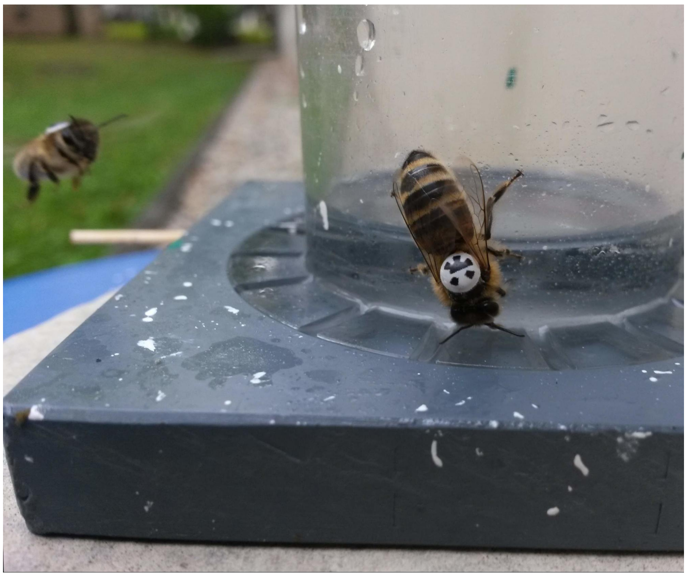

Supplementary Figure 11: **Individually marked bees at a feeding site.**

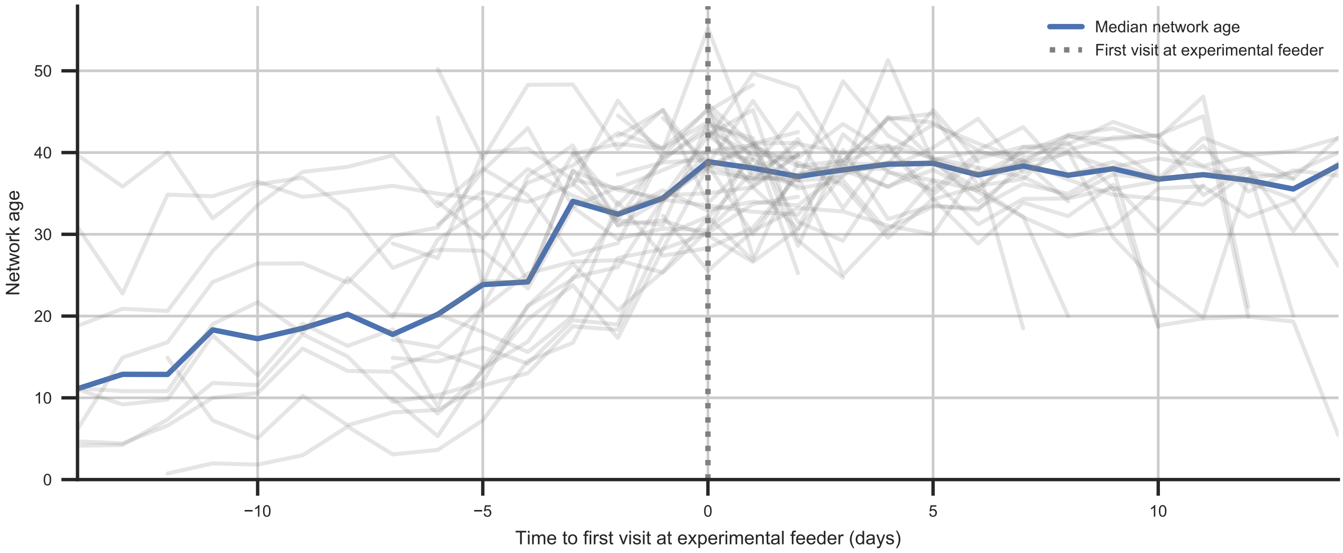

Supplementary Figure 12: **Network age development of new foragers.** For every visiting bee, their first visit to a feeding station was recorded (age 12-40 days, N=40). The plot shows the network age for these bees over the previous and next days centered on their first visit. The grey lines give the network age of individual bees and the blue line indicates the median.

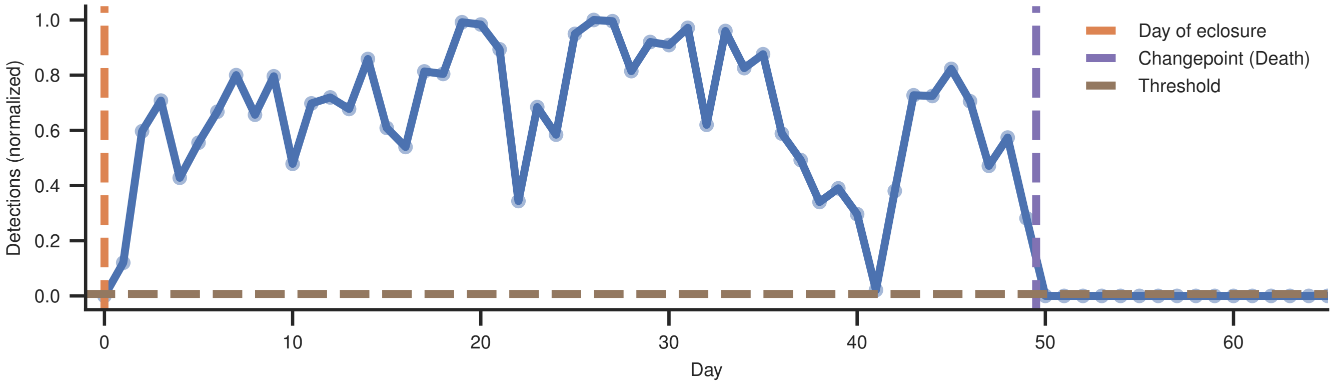

Supplementary Figure 13: **The normalized detection counts of one bee over the recording period following her introduction to the observation hive.** The time of her death (purple dotted line on the right) is the mean from N=1000 Monte Carlo samples from a Bayesian changepoint model based on optimizing a detection threshold that discriminates between days that the bee was alive and days where she was probably dead.

a
b
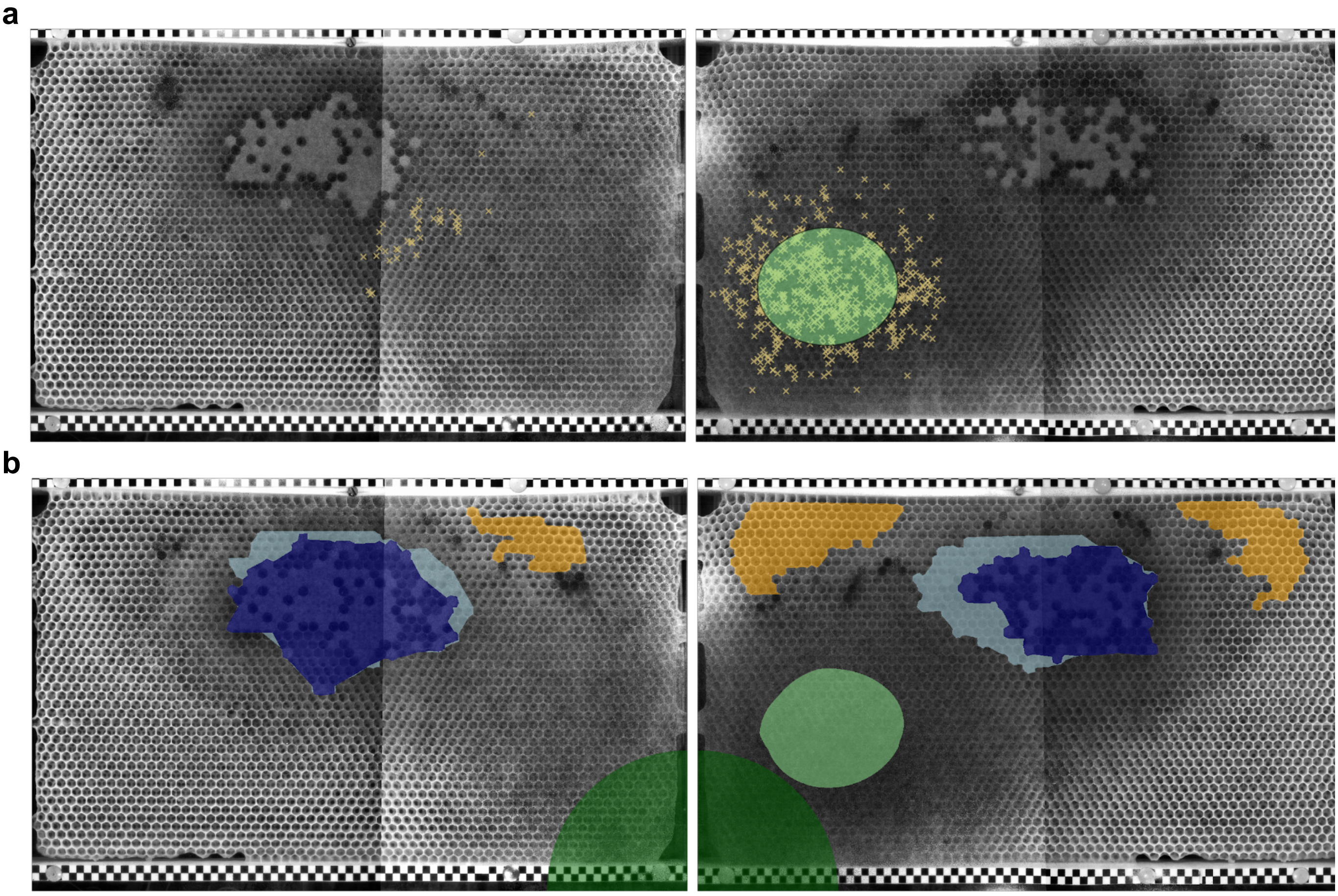

Supplementary Figure 14: **Ground truth data for the location-based task descriptors.** **a** Background-subtracted images of the two sides of the observation hive. The images from the two cameras per side have been stitched together. The markers show the location of the high-confidence waggle run detections for 2016-08-18. Nearly all are on one side of the comb. The green region indicates the dance floor location. **b** Annotations for 2016-08-18, used to generate task descriptors for every bee (dark blue: capped brood, light blue: open brood, yellow: honey, light green: dance floor, dark green: exit region).

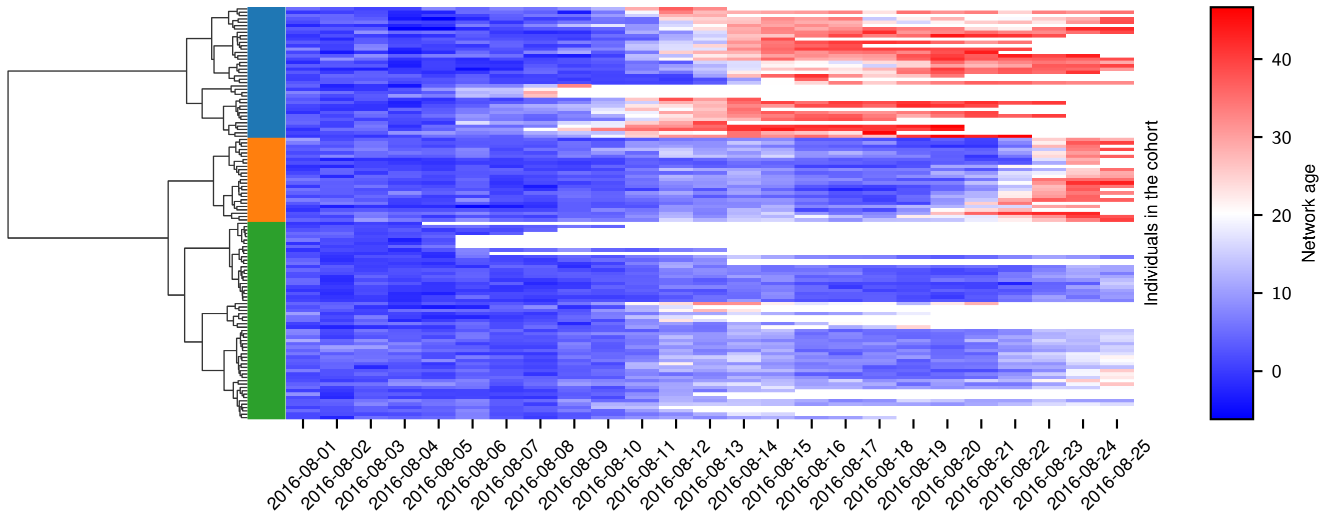

Supplementary Figure 15: **Agglomerative clustering of the network age over time of one cohort of bees (emerged on 2016-08-01)** Missing values (e.g. due to the death of the bee) are white. The three colors (blue, orange, green) on the left side correspond to the clusters (1, 2, 3) in Fig. 3b.

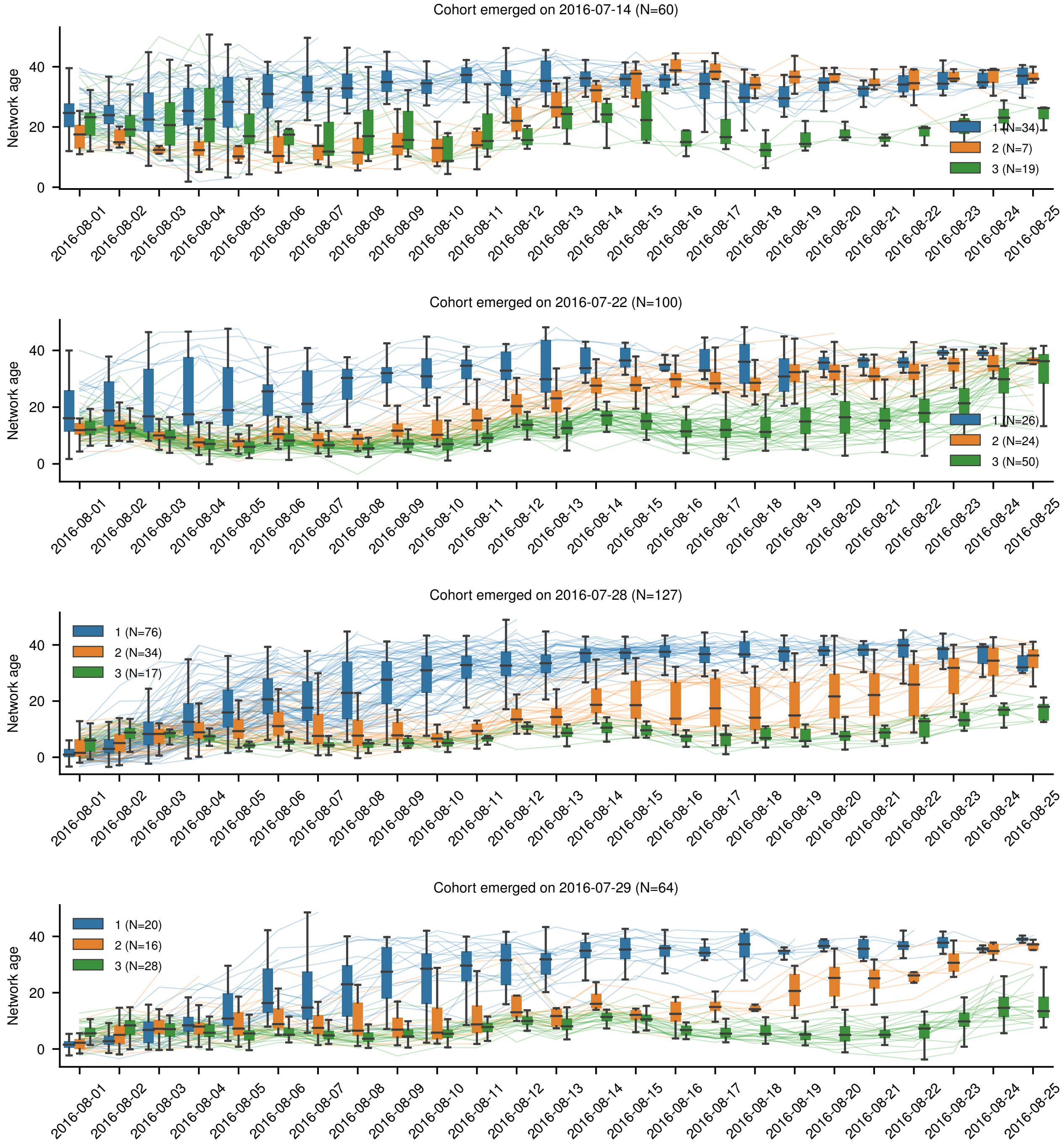

Supplementary Figure 16: **The development of the network age of different cohorts over the focal period (see individual titles for date of emergence and cohort sizes).** The individuals are grouped according to the agglomerative clustering (see legends for the number of individuals in each cohort's clusters). The lines depict the network age of the individual bees and include all data points that are summarized in the boxplots for a given day (center line, median; box limits, upper and lower quartiles; whiskers, 1.5x interquartile range).

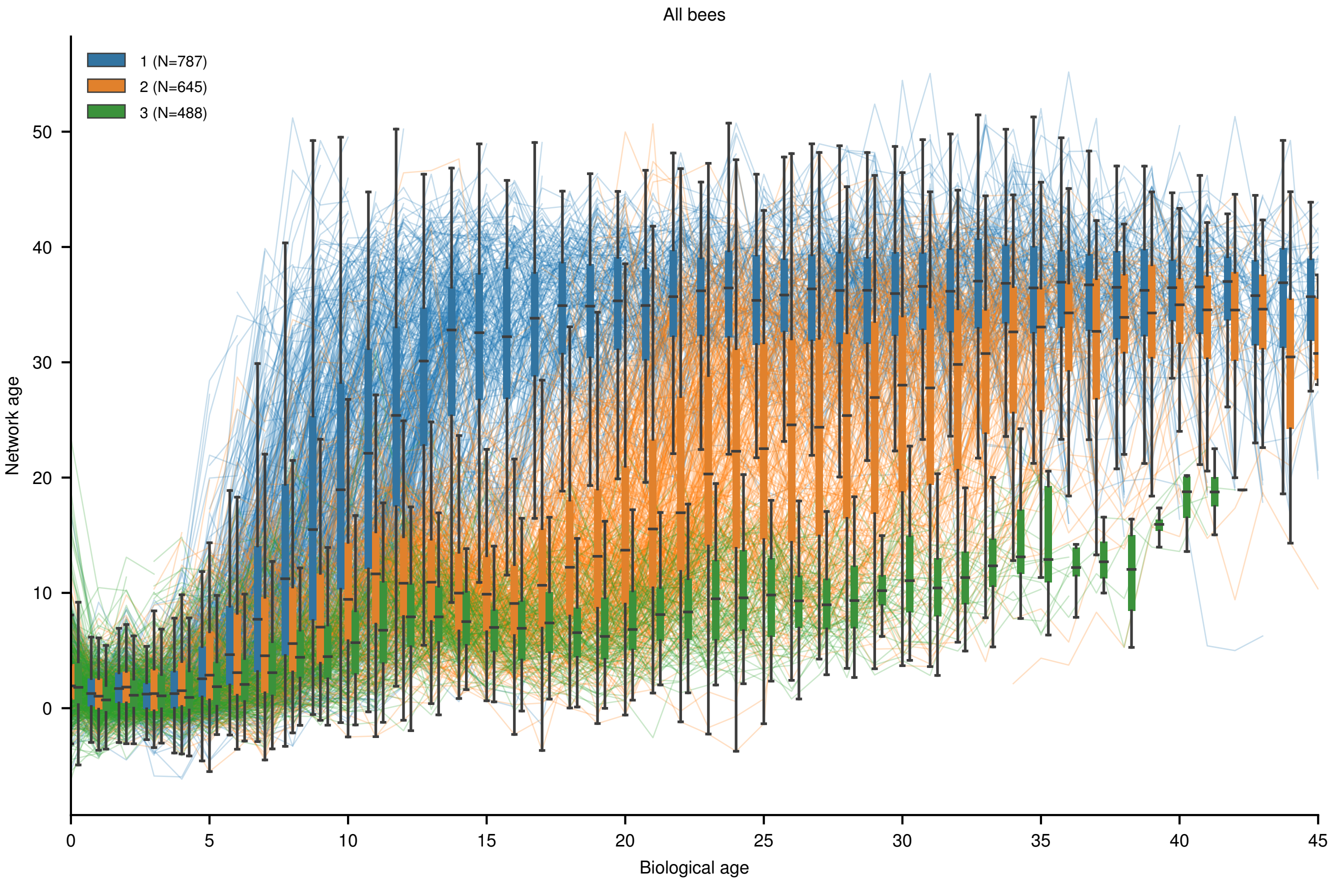

Supplementary Figure 17: **The network age development of all individuals over their age.** To calculate this, we followed a procedure analogous to clustering the cohorts but used network age per age (instead of per date) as the bees' feature vectors for all individuals (N=1920). Note that there was only a subset of 25 days of data available for each individual as some individuals were already in the hive as the focal period began and some were introduced later. The lines depict the network age of individual bees. The number of individuals in each cluster is given in the legend. The boxplots summarize bees belonging to each cluster for a given day (center line, median; box limits, upper and lower quartiles; whiskers, 1.5x interquartile range).

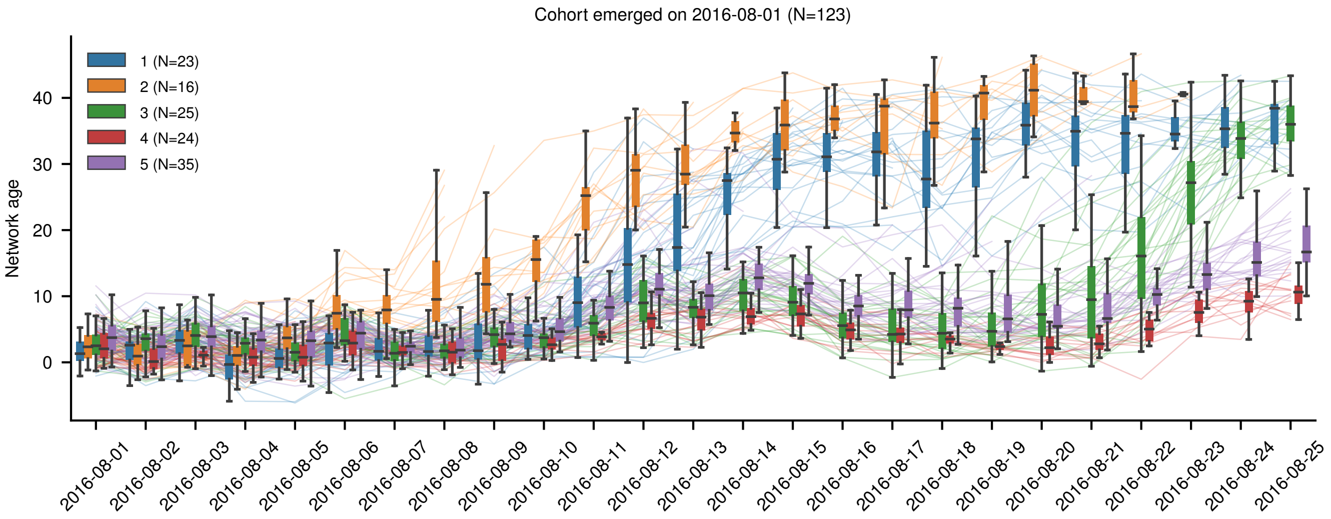

Supplementary Figure 18: **Network age development with higher number of clusters (N=5).** Cutting the dendrogram at a deeper level yields more clusters that further subdivide the previous ones. The number of individuals and the date of emergence is given in the title, the number of individuals in each cluster is given in the legend. The boxplots summarize bees belonging to each cluster for a given day (center line, median; box limits, upper and lower quartiles; whiskers, 1.5x interquartile range).

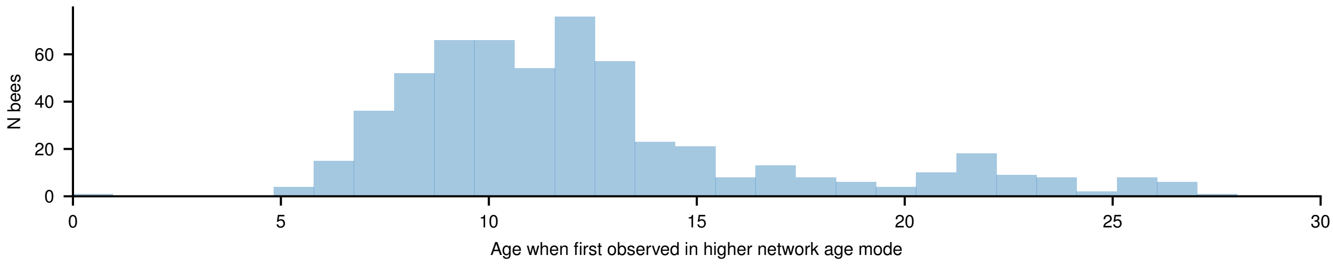

Supplementary Figure 19: **The distribution of the biological age at which a bee was first assigned to the higher network age cluster.** Starting at around age 5-7, the network age distribution becomes bimodal.

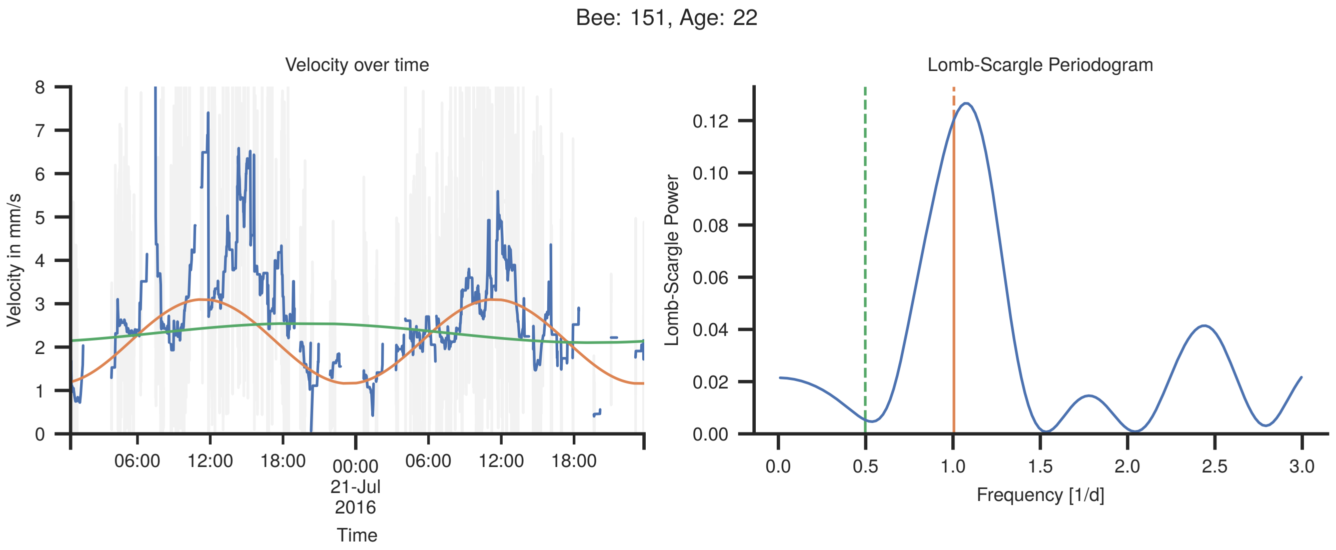

Supplementary Figure 20: **Calculation of circadian rhythmicity from movement data.** (Left) The velocity as raw data (grey line) and smoothed with a median filter (blue line) for one bee over two days. The orange sine wave has a period of one day and was fitted to the velocity data via least-squares fitting. The green line is a similar fit with a period of two days. (Right) The corresponding Lomb-Scargle periodogram with the powers at the one-day frequency (orange) and two-day frequencies (green) highlighted.

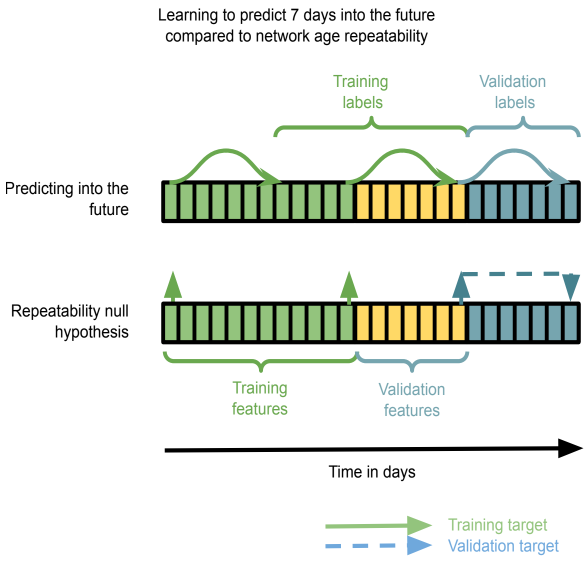

Supplementary Figure 21: **Data handling for cross-validating future predictability.** (Top) Depiction of the process of training a model based on either biological age or network age to predict the future task allocation. We make sure that we do not leak information when evaluating future predictiveness. (Bottom) Data handling for the null hypothesis to check that the predictiveness is not completely explained by the repeatability of network age and task allocation for individual bees.

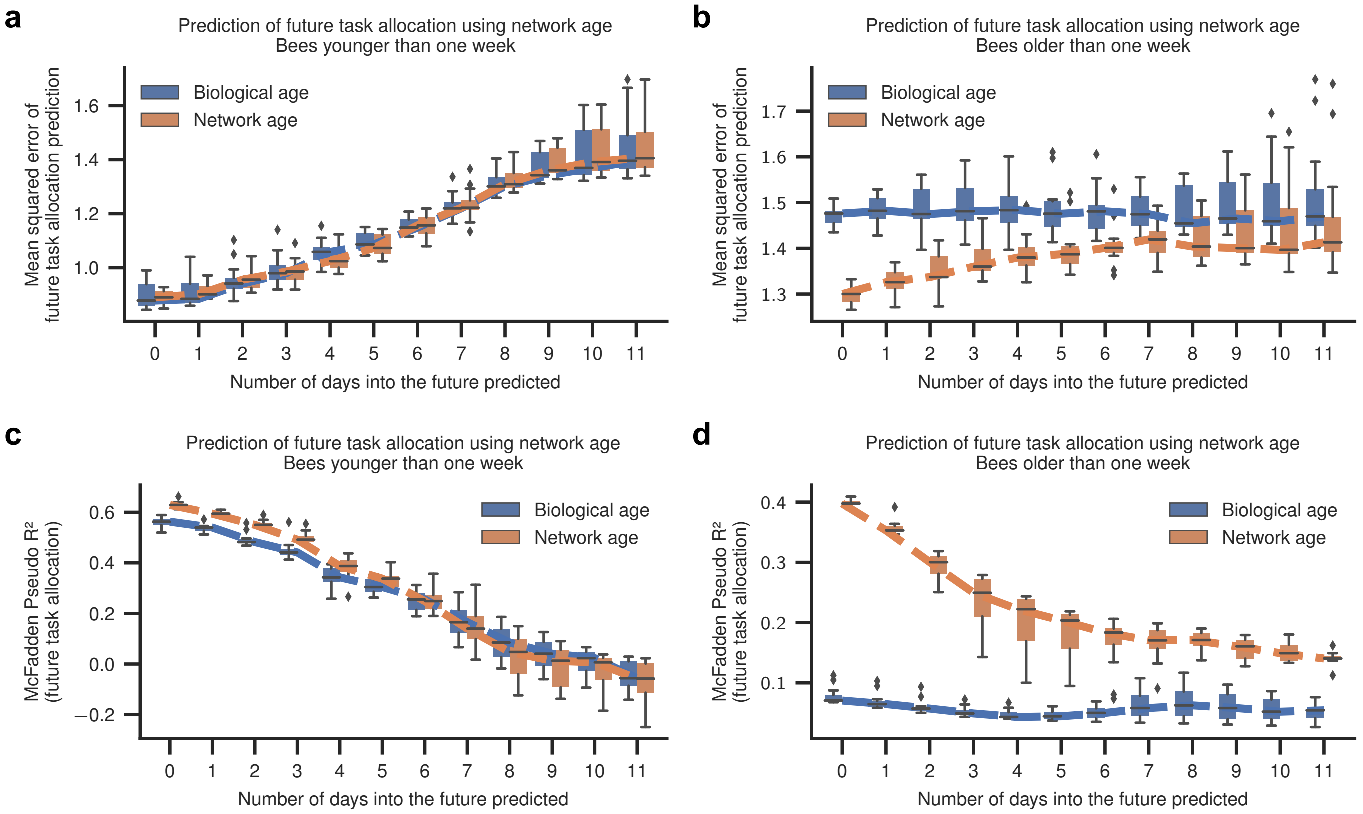

Supplementary Figure 22: **Prediction of future task allocation for young and old bees.** We distinguish between young bees (left column, < 7 days of age) and old bees (right column, > 7 days of age). **a** For young bees, we find neither biological age nor network age to be predictive. **b** For the old bees, network age consistently performs better than age. **c, d** McFadden's R^2_{McF} of the future predictability for young and old bees. Each box comprises N=12 scores from models with N=12 days of training data (center line, median; box limits, upper and lower quartiles; whiskers, 1.5x interquartile range; points, outliers).

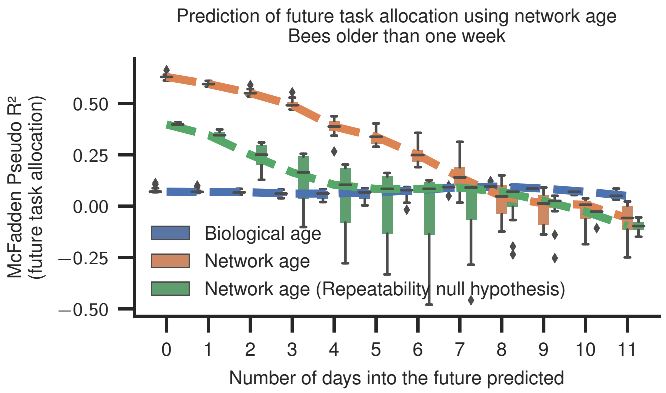

Supplementary Figure 23: **Ruling out repeatability as a cause for predictiveness of network age.** To check that the predictiveness of network age for old bees is not simply due to the bimodal distribution of the network age and its high repeatability, we compare a predictive model based on network age (orange) with the null-hypothesis that the network age of day X is sufficient to describe the coming days without explicitly modeling the changes. Each box comprises N=12 scores from models with N=12 days of training data (center line, median; box limits, upper and lower quartiles; whiskers, 1.5x interquartile range; points, outliers).

(a) R^2_{McF} scores for dependent and independent variables and models

	Days until death	Time of peak activity	Strength of circadian rhythm	Velocity (daytime)	Velocity (night)
Age	0.06 [0.06, 0.07]	0.07 [0.07, 0.08]	0.30 [0.28, 0.30]	0.12 [0.12, 0.13]	0.21 [0.21, 0.23]
Age (nonlinear)	0.07 [-1.60, 0.08]	0.08 [0.07, 0.09]	0.31 [0.29, 0.32]	0.14 [0.13, 0.15]	0.35 [0.34, 0.36]
Network age (PCA)	0.09 [0.08, 0.09]	0.06 [0.06, 0.07]	0.45 [0.43, 0.47]	0.15 [0.14, 0.16]	0.29 [0.27, 0.30]
Location (1D)	0.10 [0.10, 0.11]	0.08 [0.07, 0.08]	0.49 [0.48, 0.49]	0.17 [0.16, 0.17]	0.31 [0.30, 0.33]
Network age (PCA, nonlinear)	0.14 [0.13, 0.14]	0.08 [0.07, 0.09]	0.51 [0.50, 0.52]	0.19 [0.19, 0.20]	0.33 [0.32, 0.34]
Location (1D, nonlinear)	0.15 [0.14, 0.16]	0.09 [0.08, 0.10]	0.51 [0.50, 0.52]	0.21 [0.20, 0.22]	0.34 [0.33, 0.35]
Network age	0.16 [0.16, 0.17]	0.10 [0.09, 0.11]	0.61 [0.61, 0.62]	0.24 [0.23, 0.25]	0.32 [0.31, 0.33]
Age + Network age	0.17 [0.16, 0.17]	0.11 [0.10, 0.11]	0.61 [0.61, 0.62]	0.24 [0.23, 0.25]	0.33 [0.32, 0.34]
Location (4D)	0.21 [0.20, 0.22]	0.10 [0.09, 0.10]	0.59 [0.58, 0.59]	0.28 [0.27, 0.29]	0.32 [0.30, 0.33]
Network age (nonlinear)	0.20 [0.19, 0.20]	0.11 [0.10, 0.11]	0.63 [0.62, 0.64]	0.27 [0.26, 0.28]	0.37 [0.36, 0.38]
Network age (2D)	0.23 [0.22, 0.24]	0.11 [0.10, 0.11]	0.62 [0.61, 0.63]	0.26 [0.25, 0.27]	0.37 [0.36, 0.38]
Location (4D, nonlinear)	0.23 [0.22, 0.24]	0.10 [0.10, 0.11]	0.62 [0.61, 0.62]	0.31 [0.30, 0.31]	0.36 [0.35, 0.37]
Network age (3D)	0.23 [0.22, 0.24]	0.11 [0.10, 0.11]	0.63 [0.62, 0.63]	0.30 [0.29, 0.31]	0.37 [0.36, 0.38]
Targeted embedding (1D)	0.22 [0.21, 0.22]	0.10 [0.10, 0.11]	0.62 [0.61, 0.63]	0.30 [0.30, 0.31]	0.41 [0.40, 0.42]
Targeted embedding (1D, nonlinear)	0.23 [0.22, 0.24]	0.11 [0.10, 0.11]	0.63 [0.63, 0.64]	0.31 [0.30, 0.32]	0.41 [0.40, 0.42]
Network age (2D, nonlinear)	0.27 [0.26, 0.27]	0.12 [0.11, 0.12]	0.63 [0.63, 0.64]	0.29 [0.28, 0.30]	0.40 [0.39, 0.41]
Age + Network age (nonlinear)	0.23 [0.22, 0.23]	0.13 [0.12, 0.13]	0.65 [0.64, 0.65]	0.30 [0.29, 0.31]	0.44 [0.43, 0.45]
Network age (3D, nonlinear)	0.27 [0.26, 0.28]	0.12 [0.11, 0.13]	0.64 [0.64, 0.65]	0.33 [0.32, 0.34]	0.40 [0.39, 0.42]

(b) Improvement in R^2_{McF} (network age vs biological age)

Dependent variable	Effect size [95% CI]
Days until death	0.102 [0.093, 0.110]
Time of peak activity	0.031 [0.021, 0.040]
Strength of circadian rhythm	0.317 [0.308, 0.329]
Velocity (daytime)	0.116 [0.108, 0.127]
Velocity (night)	0.107 [0.093, 0.121]

(c) Improvement in R^2_{McF} (network age vs targeted embeddings)

Dependent variable	Effect size [95% CI]
Days until death	0.050 [0.042, 0.061]
Time of peak activity	0.000 [-0.009, 0.011]
Strength of circadian rhythm	0.009 [0.000, 0.019]
Velocity (daytime)	0.065 [0.052, 0.077]
Velocity (night)	0.083 [0.072, 0.095]

(d) Improvement in R^2_{McF} (network age vs location (1D)))

Dependent variable	Effect size [95% CI]
Days until death	0.061 [0.052, 0.069]
Time of peak activity	0.025 [0.016, 0.034]
Strength of circadian rhythm	0.127 [0.119, 0.138]
Velocity (daytime)	0.073 [0.061, 0.083]
Velocity (night)	0.008 [-0.007, 0.022]

Supplementary Table 1: R^2_{McF} values for different models when predicting different behavior-related metrics. Biological age consistently performs worse than predictions based on network age. Our method can also be applied without spatial information by optimizing the CCA for these metrics, yielding embeddings that are strongly predictive of them while still only using information available from the social networks (see 'Targeted embedding (1D)'). (Bottom) 95% bootstrap confidence intervals of the effect sizes (b) R^2 values for network age and biological age, c) R^2_{McF} values for targeted embeddings and network age, N=128).

	Brood area	Dance floor	Honey storage	Near exit	Combined
Age	0.46 [0.45, 0.47]	0.28 [0.27, 0.29]	0.00 [0.00, 0.00]	0.32 [0.31, 0.33]	0.34 [0.34, 0.35]
Age (nonlinear)	0.51 [0.50, 0.52]	0.35 [0.34, 0.36]	0.09 [0.09, 0.10]	0.41 [0.40, 0.42]	0.38 [0.38, 0.39]
Network age (1% of bees tagged)	0.58 [0.07, 0.79]	0.50 [0.08, 0.68]	0.03 [0.00, 0.21]	0.49 [0.12, 0.73]	0.52 [0.13, 0.70]
Network age (Only euclidean proximities)	0.51 [0.50, 0.52]	0.37 [0.36, 0.38]	0.17 [0.16, 0.18]	0.53 [0.52, 0.54]	0.52 [0.51, 0.52]
Network age (Only euclidean proximities, nonlin...	0.60 [0.60, 0.61]	0.55 [0.54, 0.56]	0.20 [0.18, 0.21]	0.56 [0.55, 0.57]	0.55 [0.55, 0.56]
Network age (1% of bees tagged, nonlinear)	0.62 [0.08, 0.81]	0.58 [0.16, 0.73]	0.16 [0.03, 0.34]	0.55 [0.17, 0.77]	0.55 [0.19, 0.72]
Network age (5% of bees tagged)	0.76 [0.66, 0.83]	0.61 [0.52, 0.68]	0.03 [0.00, 0.08]	0.60 [0.52, 0.69]	0.65 [0.58, 0.70]
Network age (PCA)	0.80 [0.79, 0.80]	0.53 [0.52, 0.54]	0.00 [0.00, 0.00]	0.58 [0.57, 0.59]	0.65 [0.64, 0.65]
Network age (PCA, nonlinear)	0.80 [0.80, 0.80]	0.61 [0.60, 0.62]	0.27 [0.26, 0.28]	0.66 [0.65, 0.66]	0.66 [0.66, 0.67]
Network age (Only proximity events)	0.79 [0.79, 0.80]	0.64 [0.64, 0.65]	0.03 [0.03, 0.03]	0.60 [0.59, 0.60]	0.67 [0.67, 0.68]
Network age (5% of bees tagged, nonlinear)	0.77 [0.63, 0.83]	0.66 [0.58, 0.73]	0.24 [0.15, 0.32]	0.67 [0.57, 0.73]	0.67 [0.59, 0.72]
Network age	0.80 [0.80, 0.81]	0.63 [0.63, 0.64]	0.03 [0.03, 0.04]	0.61 [0.60, 0.62]	0.68 [0.68, 0.69]
Age + Network age	0.81 [0.80, 0.81]	0.63 [0.63, 0.64]	0.10 [0.09, 0.11]	0.61 [0.60, 0.62]	0.69 [0.69, 0.69]
Network age (Only proximity events, nonlinear)	0.81 [0.80, 0.81]	0.68 [0.67, 0.69]	0.32 [0.31, 0.32]	0.68 [0.67, 0.69]	0.70 [0.69, 0.70]
Network age (nonlinear)	0.82 [0.81, 0.82]	0.68 [0.67, 0.69]	0.33 [0.32, 0.33]	0.69 [0.69, 0.70]	0.71 [0.70, 0.71]
Age + Network age (nonlinear)	0.82 [0.82, 0.83]	0.69 [0.68, 0.69]	0.36 [0.35, 0.37]	0.70 [0.69, 0.70]	0.72 [0.71, 0.72]
Network age (2D)	0.84 [0.84, 0.85]	0.65 [0.64, 0.65]	0.46 [0.45, 0.46]	0.64 [0.63, 0.64]	0.73 [0.73, 0.74]
Network age (2D, nonlinear)	0.85 [0.85, 0.85]	0.69 [0.69, 0.70]	0.49 [0.48, 0.50]	0.70 [0.70, 0.71]	0.74 [0.74, 0.75]
Network age (3D)	0.84 [0.84, 0.85]	0.71 [0.70, 0.71]	0.46 [0.45, 0.47]	0.70 [0.70, 0.71]	0.76 [0.75, 0.76]
Network age (3D, nonlinear)	0.86 [0.85, 0.86]	0.75 [0.74, 0.75]	0.50 [0.49, 0.51]	0.76 [0.76, 0.77]	0.77 [0.77, 0.77]

Supplementary Table 2: R^2_{McF} median scores and bootstrapped 95% CI for different combinations of independent variables in the rows and dependent variables in the columns. We evaluated the following independent variables: different dimensionalities of network age ('Network age', 'Network age (2D)', 'Network age (3D)', see Methods: Network age - CCA), network age derived without supervision ('PCA', see Methods: Network age - PCA), with a subsample of the bees ('1% of bees tagged' and '5% of bees tagged', see Supplementary Note 2.2), and based only on spatial proximity ('Euclidean proximities' and 'Proximity events'). The likelihood ratio test was evaluated for the combined models (e.g. 'Age + Network age', see Methods: Statistical comparison of models).

Feeder	GPS coordinate	Distance from hive
F1	52.455726, 13.294224	217 m
F2	52.454866, 13.293092	338 m
F3	52.454547, 13.291737	430 m
F4	52.453682, 13.292841	451 m

Supplementary Table 3: GPS coordinates and distances from the hive for the feeders used in the forager group experiment.

Date	Location	Forager bees observed at feeder
2016-08-01	Hive-F1	135, 199, 217, 220, 228, 233, 253, 319, 392, 539, 727, 818, 845, 860, 1110
2016-08-02	F1	135, 217, 233, 253, 648, 845, 860
2016-08-03	F1	135, 228, 233, 253, 392, 648, 845, 860
2016-08-04	F1	220, 228, 233, 392, 648, 710, 885
2016-08-05	F1	135, 220, 228, 233, 392, 644, 648, 710, 769, 885, 931
2016-08-08	F1-F2	199, 319, 337, 392, 555, 644, 710, 769, 885, 1593
2016-08-09	F1-F2	199, 319, 337, 392, 555, 644, 648, 710, 769, 785, 885, 912, 1122, 1362, 1518, 1593, 2031
2016-08-10	F1-F2	199, 319, 337, 392, 648, 769, 885, 1122, 1362, 1593, 2031
2016-08-11	F2	199, 319, 337, 392, 644, 769, 785, 885, 1122, 1362, 1518, 1593, 2031
2016-08-12	F2	199, 319, 337, 392, 769, 785, 885, 1122, 1362, 1518, 1593, 2031
2016-08-14	F2	199, 319, 337, 392, 555, 644, 710, 769, 785, 885, 1232, 1362, 1518, 1593, 2031
2016-08-16	F2	199, 319, 337, 710, 769, 885, 912, 1122, 1362, 1518, 1593, 2031
2016-08-17	F2	199, 319, 337, 644, 710, 769, 885, 912, 1122, 1518, 1593, 2031
2016-08-18	F2	199, 319, 337, 644, 769, 885, 912, 1122, 1518, 2031
2016-08-19	F2	319, 710, 885, 1593, 2031
2016-08-22	F2-F3	319, 644, 710, 885, 1593, 2031
2016-08-23	F3	1180, 1197, 1232, 1471, 1593, 1662, 1714, 1799, 2031, 2106, 2984
2016-08-24	F3	1180, 1197, 1232, 1471, 1593, 1714, 1793, 1799, 2031, 2106, 2984
2016-08-25	F4	1180, 1197, 1714, 1793, 1799, 2031, 2106

Supplementary Table 4: Tag IDs and locations observed forager bees during the forager group experiment.

References

[1] Seeley, T. D. Adaptive Significance of the Age Polyethism Schedule in Honeybee Colonies.*Behavioral Ecology and Sociobiology***11**, 287–293 (1982).

[2] Wario, F., Wild, B., Couvillon, M. J., Rojas, R. & Landgraf, T. Automatic methods for long-term tracking and the detection and decoding of communication dances in honeybees. *Frontiers in Ecology and Evolution* **3** (2015).
